# Nurses' Earnings

**Published:** 1887-12-10

**Authors:** 


### Nurses' Earnings.

I am glad to see that the question of nurses receiving their own earnings has been raised, and I think the sooner arrangements are made for altering private nurses' pay the better. In the private nursing institutions it is a great mistake not to let the nurses share in the profits, instead of giving them only a stated salary. If committees would fix the salary at a somewhat lower rate (say £20 per annum), and then give a certain sum per week for each week's employment, their institutions would be none the poorer, and the nurses would be more anxious to keep their cases, more willing to be always employed, and the arrangement would encourage those diligent workers who sincerely strive to do their duty. Some such plan, I feel sure, would tend to unite the interests of the nurses with those of their institutions, whereas by the fixed salary system that is far from being the case. A Trained Nurse.

